# Assessing the role of adolescent hormonal contraceptive use on risk for depression: a 3-year longitudinal study protocol

**DOI:** 10.1186/s12905-022-01623-2

**Published:** 2022-02-23

**Authors:** Bita Zareian, Christine Anderl, Joelle LeMoult, Liisa A. M. Galea, Jerilynn C. Prior, Jason D. Rights, Colin J. Ross, Sabrina Ge, Annie C. Hayward, Frances S. Chen

**Affiliations:** 1 Department of Psychology, University of British Columbia, Vancouver, Canada; 2 Leibniz-Institut für Wissensmedien, Tübingen, Germany; 3 Djavad Mowafaghian Centre for Brain Health, University of British Columbia, Vancouver, Canada; 4 Centre for Menstrual Cycle and Ovulation Research, Endocrinology and Metabolism, University of British Columbia, Vancouver, Canada; 5 BC Women’s Health Research Institute, Vancouver, Canada; 6 School of Population and Public Health, University of British Columbia, Vancouver, Canada; 7 BC Children’s Hospital Research Institute, Vancouver, Canada; 8 Faculty of Pharmaceutical Sciences, University of British Columbia, Vancouver, Canada; 9 Department of Medicine, Faculty of Medicine, University of British Columbia, Vancouver, Canada

**Keywords:** Birth control, Stress reactivity, Cortisol, Puberty, Emotional development, Social functioning, Major depressive disorder, Mood disorder, Mental health

## Abstract

**Background:**

The incidence of depression in human females rises steadily throughout adolescence, a critical period of pubertal maturation marked by increasing levels of gonadal hormones including estrogens and progesterone. These gonadal hormones play a central role in social and emotional development and may also contribute to the increased occurrence of depression in females that begins in early adolescence. In this study, we examine whether and how introducing synthetic estrogen and progestin derivatives through the use of combined hormonal contraceptives (CHC), affects adolescent females’ risk for developing depression. We further assess potential links between CHC use and alterations in stress responses and social-emotional functioning.

**Methods:**

Using a longitudinal cohort design, we will follow a sample of adolescent females over the span of three years. Participants will be assessed at three time points: once when they are between 13 and 15 years of age, and at approximately 18 and 36 months after their initial assessment. Each time point will consist of two online sessions during which participants will complete a clinical interview that screens for key symptoms of mental health disorders, along with a series of questionnaires assessing their level of depressive symptoms and history of contraceptive use. They will also complete a standardized social-evaluative stress test and an emotion recognition task, as well as provide saliva samples to allow for assessment of their circulating free cortisol levels.

**Discussion:**

In this study we will assess the effect of CHC use during adolescence on development of Major Depressive Disorder (MDD). We will control for variables previously found to or proposed to partially account for the observed relationship between CHC use and MDD, including socioeconomic status, age of sexual debut, and CHC-related variables including age of first use, reasons for use, and its duration. In particular, we will discover whether CHC use increases depressive symptoms and/or MDD, whether elevated depressive symptoms and/or MDD predict a higher likelihood of starting CHC, or both. Furthermore, this study will allow us to clarify whether alterations in stress reactivity and social-emotional functioning serve as pathways through which CHC use may result in increased risk of depressive symptoms and/or MDD.

**Supplementary Information:**

The online version contains supplementary material available at 10.1186/s12905-022-01623-2.

## Background

Depression is a debilitating mental disorder that afflicts more than 260 million individuals worldwide each year [1]. Human females are nearly two times more likely than males to develop depression [2], and the increased incidence of depression in females rises steadily throughout adolescence [3]. During adolescence, young females undergo a critical period of pubertal maturation marked by increasing levels of estrogens, initially, and eventually of progesterone, gonadal hormones that have been shown to affect both the function and structure of brain regions responsible for emotional processing [4]. Thus, changes in gonadal hormone levels may play a role in females’ social and emotional experiences in the short-term and in the long run, thereby potentially contributing to the increased occurrence of depression in females in early adolescence and throughout the transition into adulthood [5]. In this study, we examine whether and how introducing a supra-physiological dose of synthetic estrogens and approximately physiological dose of progestin derivatives through the use of combined hormonal contraceptives (CHC), affects female adolescents’ risk for developing depressive symptoms, as well as their risk for receiving a diagnosis of major depressive disorder (MDD). We further assess potential links between CHC use and alterations in stress responses and social-emotional functioning, and the possible mediating effects of stress responses and social-emotional functioning in the association between CHC use and depression.

Recent data from North America suggest that the prevalence of CHC use among females between the ages of 15–19 is approximately 20–30% ([6] (current prevalence); [7] (past month prevalence); [8] (current prevalence)). Worldwide estimates for use of CHC by adolescent females range from 10 to 30% [7, 9, 10]. Despite the high CHC use by adolescent females, however, it is still unclear precisely how exposure to synthetic estrogens and progesterone derivatives and the ensuing effects on endogenous gonadal hormone profiles affect young females’ social and emotional development [11].

Growing evidence suggests that CHC use in adolescents is associated with increases in depressive symptoms in both the short-term [12–14] and the long run [15, 16]. In a cross-sectional study, Lindberg et al. [13] found that CHC use in adolescence was associated with higher likelihood of concurrent antidepressant use. Anderl et al. [15] found that CHC use during adolescence (assessed via retrospective self-report) was associated with higher likelihood of being diagnosed with MDD in adulthood. Furthermore, in three distinct analyses of two large prospective cohort studies, CHC use in adolescents was associated with increased odds of first use of antidepressants [14], experiencing depressive symptoms [12], and first diagnosis of MDD, both while still using CHC [12] and in adulthood, years later [16]. These associations held even after controlling for variables that have previously been proposed to account for the association between CHC use and depression, such as the age of sexual debut and the age of first CHC use [12, 15, 16]. Given these patterns of findings, it is critical for research to address how and why CHC use during adolescence may contribute to an increased risk of depression.

## Proposed primary mechanisms of action

Although the exact effect of CHCs on female adolescent social and emotional development is unclear [11], previous findings suggest that the effects of gonadal hormones on conferring vulnerability for depression may partly be mediated by gonadal hormone-dependent modulation of (a) hypothalamic pituitary adrenal (HPA) axis regulation [5, 17, 18], and (b) social-emotional functioning [19]. Indeed, CHC use suppresses natural production of gonadal hormones [20] integral to the development and maturation of various brain regions involved in response to stress and social cues [21, 22]. Atypical responses to stress and social cues, in turn, have been linked to development of depression [23, 24]. Thus, HPA axis dysregulation and social dysfunction represent two potential pathways through which CHC use could result in higher vulnerability for depression.

### HPA axis regulation

The HPA axis is involved in the secretion of adrenal hormones, including cortisol, and plays a crucial role in the body’s response to stress [25]. Although there is limited research on how gonadal hormones influence HPA axis regulation in human adolescents, estradiol increases HPA axis activity in adolescent rats [26, 27]. This increase in HPA axis activity in puberty alters the reactivity of corticosterone, the main glucocorticoid in rodents, in response to stress in rodents [28, 29]. Although it is unclear how these findings translate to human adolescents, levels of circulating gonadal hormones, as well as general increases of these hormones during puberty, have both been associated with responses to stress in humans [21].

Several studies have shown an aberrant cortisol response to stress in CHC users [30–33]. For instance, Bouma et al. [30] found that, in contrast to naturally cycling female adolescents, adolescent CHC users showed no cortisol reactivity in response to an in-lab social stress test. Furthermore, Sharma et al. [33] found that adult females who had continuously used CHC since puberty showed blunted stress responses to a similar social stress test, the Trier Social Stress Test (TSST) [34], compared to females who had continuously used CHC only since adulthood. Studies in adult females also showed blunted cortisol reactivity in response to a variant of the TSST in CHC users [31, 32].

In addition, CHC use may affect the normal diurnal pattern for cortisol. Bouma et al. [30] found that adolescent female CHC users had a blunted cortisol awakening response (CAR), in comparison to their naturally cycling counterparts. Furthermore, in comparison to naturally cycling adult females, adult CHC users had higher levels of awakening cortisol [32] and higher mean levels of circulating cortisol than non-CHC users [35, 36]. Blunted cortisol reactivity to stress [37], atypical cortisol awakening responses [38], and elevated levels of cortisol throughout the day have all been associated with depression [24]. These data suggest that altered HPA axis responses may be one pathway through which CHC use could result in depression.

### Social-emotional functioning

Similar to the HPA axis, social-emotional functioning undergoes extensive changes during adolescence and is affected by fluctuations in gonadal hormones [22]. For example, adolescence is the life phase during which humans develop an advanced capacity to mentalize (consider others’ mental states) and empathize (understand other’s emotional state); both of which are required for forming mature social relationships [19, 22].

The ability to accurately detect and respond to others’ facial expressions of emotion is a core component underlying the capacity to mentalize and empathize. The speed and accuracy of emotion detection have both been linked to natural fluctuations of gonadal hormones across the menstrual cycle [39, 40] as well as to CHC use [41]. Findings on specific emotions have not been entirely consistent across studies, potentially due, in part, to methodological differences (*e.g*., whether static images or dynamic videos are used, and the complexity of the emotions being expressed). Overall, however, CHC use has been linked to relative deficits in emotion recognition as well as attentional biases towards negative emotional expressions (41). Given that depression has been linked to altered sensitivity to certain emotional facial expressions (*e.g.*, heightened sensitivity to sad faces and decreased sensitivity to happy faces) [42, 43], another pathway through which CHC use could lead to an increased risk for depression is through its effect on adolescent females’ social-emotional functioning, and specifically, on emotion recognition.

## Secondary mediators, moderators, and outcomes

In order to gain a more complete picture of adolescents’ development and responses to CHC use, we will explore additional potential mechanisms of action, moderators, and outcomes. First, we will assess the role of reward processing, using the behavioral model of signal detection [44, 45]. Reward processing is affected by changes in gonadal hormone levels during puberty [46]; furthermore, females’ behavioral responses to rewards vary along with natural fluctuations of estrogens and progesterone during the menstrual cycle [47]. Given that reward responsiveness is also linked to anhedonia [48–50], one of the cardinal symptoms of depression characterized by reduced motivation or ability to experience pleasure, changes in reward processing may mediate the association between CHC use and depression risk.

Second, we will assess whether and how the use of CHC predicts changes in additional components of social-emotional functioning, including social decision-making [51, 52], and perceived quality of social relationships (*e.g.*, perceived social support, and perceived loneliness) [53, 54]. Finally, we will explore whether and how genetic variants—including those involved in the uptake and metabolism of the synthetic estrogen and progestin derivatives in CHC [55, 56], or previously linked to risk for depressive symptoms [55]—moderate the predicted relationship between CHC use and depression risk.

## Study objectives

Our first aim is to establish whether and how adolescent CHC use is associated with current and future depressive symptoms, as well as a current and future diagnosis of MDD. We hypothesize that CHC use will be associated with higher levels of depressive symptoms both concurrently and longitudinally, and will increase the likelihood of concurrent and prospective MDD diagnosis. Our second aim is to investigate links between CHC use and alterations in HPA axis regulation and social-emotional functioning. We hypothesize that adolescent CHC use predicts lasting alterations in HPA axis regulation (*i.e.*, cortisol reactivity and recovery in response to an acute stressor, CAR, and diurnal cortisol release) and social-emotional functioning (i.e., emotion recognition). Our third aim is to examine the role of HPA axis regulation and social-emotional functioning in the context of CHC use and depression. We hypothesize that HPA axis regulation and social-emotional functioning mediate the relationship between CHC use and depression. Finally, on an exploratory basis, we will examine whether CHC use predicts changes in reward processing, social decision making, and perceived social support and loneliness. We will also examine the potential role of changes in reward processing and social decision-making in mediating the hypothesized relationship between CHC use and depression risk, and the potential moderating role of genetic variants in the hypothesized relationship between CHC use and depression risk.

## Methods

### Study design and setting

This is a longitudinal cohort study which will follow a sample of female adolescents over the span of three years. Participants will be assessed at three timepoints: once when they are between 13–15 years of age (Wave 1), then approximately 18 months (Wave 2), and approximately 36 months (Wave 3) after their initial assessment. We chose to follow adolescents of this age over three years in order to capture a developmental window in which many North American adolescents begin using CHCs [6–8], and experience their first episode of MDD [3]. Each wave will consist of two online sessions as well as an at-home saliva sampling protocol to assess participants’ circulating cortisol levels. At the end of each session, participants will be reimbursed for their participation in the study. Testing sessions prior to March 2020 (prior to the widespread outbreak of COVID-19) were conducted in person, which included 14 participants who participated in Wave 1. For all of the remaining testing sessions, all three waves will be conducted online using a videoconferencing platform.

### Participants

We aim to recruit a convenience sample of 320 post-menarcheal adolescents aged 13–15 who were assigned female at birth, and the parent/guardian who is their primary caregiver. Recruitment efforts began in September 2019 and included local media appearances and online advertisements on Facebook and Instagram, as well as poster advertisements in community centers, libraries, coffee shops, and medical clinics. Exclusion criteria for all participants at Wave 1 included factors that may substantially impact gonadal and cortisol hormone levels or impede an individual’s ability to fully participate in the study: (1) the onset of menarche at younger than ten years of age, (2) any current or past medical conditions that affect gonadal hormone levels (*e.g.*, endometriosis, pregnancy in the past 12 months), (3) meeting the DSM-5 diagnostic criteria for substance use disorder, mania, or psychosis, (4) head trauma in the past year that resulted in unconsciousness for more than 5 min, or a life-time history of head trauma that resulted in unconsciousness for more than 60 min, (5) previous participation in studies using the same social stress task as the one used in this study, (6) the adolescent and/or participating parent/guardian not being fluent in English, and finally (7) the participant and/or participating parent/guardian having a close prior personal or professional relationship with the KSADS-PL interviewer (see below).

### Procedure

All study procedures were approved by the University of British Columbia Behavioural Research Ethics Board (H18-00961, March 4th, 2021). Interested parents/guardians will first complete a brief phone screening interview to assess their family’s eligibility for participation in the study. Ineligible families will be thanked for their time and will receive a list of community mental health resources. Eligible participants and their parents/guardians will be invited and scheduled for a videoconference session during the early follicular phase of their menstrual cycle (i.e., cycle days 1 to 5). During the *first videoconference session* both the parent/guardian and adolescent will complete a clinical interview with a graduate student supervised by a registered clinical psychologist. The adolescent will complete a reward processing task, report on their hormonal contraceptive use and complete a battery of questionnaires assessing depression severity, social support, level of loneliness, health and quality of life, pubertal stage, and history of exposure to stress and traumatic experiences. The adolescent will also be asked about their gender identity, age of sexual debut, and history of pregnancy. Finally, the adolescent will be asked to measure their height and weight during the videoconferencing session under the guidance of a research assistant. If the adolescent meets all the eligibility criteria, they will be asked to provide a saliva sample for DNA testing, and will be invited for a *second videoconference session,* scheduled to occur while they are still in the follicular phase of the same menstrual cycle.

In the *second videoconference session*, adolescents in the early follicular phase will complete an emotion recognition task. They will also complete questionnaires assessing sociodemographic status and medical history. Participants will be asked to have any medications, vitamins and supplements that they are currently taking close by, to aid in answering questions about their current and recent past history of medication use. Finally, participants will undergo an acute social stressor during which they will provide saliva samples (for later analysis of cortisol levels) and report on their stress levels before, during, and after the stressor. Participating parents/guardians will also be asked to complete a questionnaire about their sociodemographic status and their child’s medical history.

On two of the remaining three days of the adolescent’s early follicular phase, participants will be asked to collect saliva samples, five times per day (three times in the morning, once after school and once before bedtime). Participants will receive automated email reminders for each saliva sample, and will also be asked to complete a survey after each saliva sample, asking them about their compliance with the saliva sampling instructions. These samples will be used to assess the diurnal cortisol rhythm. Participants are instructed to store their saliva samples in their home freezers immediately after collection, and to return the samples to us as soon as possible. Once returned to the lab, the saliva samples will be kept in a low-temperature freezer (− 30 °C or lower) until analysis.

Participants and their parents/guardians will be invited to complete the same procedures 18 months (Wave 2) and 36 months (Wave 3) after their first session (See Table 1). All procedures will be repeated in subsequent waves, with the exception of the CTQ-SF and DNA sampling that will only be administered in the first wave. For the social stressor task, we will use different arithmetic and story prompts in each wave to minimize repeated testing effects. To promote participant retention across all three waves, we are providing participants with nominal tokens of appreciation (e.g., logoed gifts) and maintaining regular contact with participating families by sending newsletters and holiday cards throughout the year.

**Table 1:** Timing of assessments

		Wave 1			Wave 2			Wave 3		
	Assessment	S1	S2	At-home	S1	S2	At-home	S1	S2	At-home
HC use	HC use questionnaire	✓			✓			✓		
Depression	K-SADS-PL	✓			✓			✓		
	CDI-SF	✓			✓			✓		
HPA axis regulation	TSST-C		✓			✓			✓	
	Diurnal cortisol			✓			✓			✓
Social-emotional functioning	Emotion recognition task		✓			✓			✓	
	CASSS	✓			✓			✓		
	UCLA loneliness scale	✓			✓			✓		
	Ultimatum Game		✓			✓			✓	
Reward sensitivity	Probabilistic reward task	✓			✓			✓		
Genetic	Genetic testing	✓								
Covariates and control variables	Sociodemographic & medical history data		✓			✓			✓	
	CTQ-SF	✓								
	PSS		✓		✓			✓		
	SF-36	✓			✓			✓		
	Tanner staging	✓			✓			✓		

### Measures

#### Hormonal contraceptive use

A hormonal contraceptive use questionnaire was created for this study based on the United States National Health and Nutrition Examination Survey and Danish Sex Hormone Register Study, and is used to assess participants’ history of hormonal contraceptive use (see Additional file 1). At all waves, participants will be asked about their current use of hormonal contraceptives and whether they have plans to use them within the next month. In addition, at Wave 1, if participants indicate that they have previously used hormonal contraceptives, they will be asked about their age at the start of use, types of hormonal contraceptives used, overall length of time of hormonal contraceptive use, and their reasons for using and discontinuing use of hormonal contraceptives. At Waves 2 and 3, we will ask whether their status of hormonal contraceptive use has changed since their previous session, and will obtain detailed information about any change in the CHC use between the two sessions.

#### Assessment of depression

The Kiddie Schedule for Affective Disorders and Schizophrenia for School-Age Children – Present and Lifetime (KSADS-PL) [57] is a semi-structured clinical interview designed to screen for key symptoms from past and current episodes of DSM disorders, and is used in this study to screen for the diagnosis of (1) major depressive disorder (MDD), (2) mental health disorders most commonly comorbid with MDD such as generalized anxiety disorder (GAD), and (3) mental health disorders that disqualify participants from participating in this study (i.e., mania, psychosis, and substance use disorder). Additional supplementary sheets for each diagnostic area are administered when a participant screens positive for a key symptom. To ensure an accurate clinical assessment of the participant, both the adolescent and the participating parent/guardian will be interviewed separately by the same graduate student. All interviewers will be trained and supervised by a registered clinical psychologist. This study will use the most recent KSADS-PL, adapted to meet the DSM-5 criteria [58]. At Wave 1, we will screen for MDD, persistent depressive disorder, bipolar disorder, GAD, panic disorder, social anxiety disorder, separation anxiety disorder, phobias, agoraphobia, obsessive compulsive disorder, eating disorders, substance use disorders, alcohol use disorder, post-traumatic stress disorder, schizophrenia spectrum and other psychotic disorders. At Waves 2 and 3, we will screen for only MDD, persistent depressive disorder, and eating disorders. The KSADS-PL has been shown to have good inter-rater reliability, construct validity and predictive validity in children and adolescents [59, 60].

The children’s depression inventory–short form (CDI-SF) [61] is a 10-item scale that measures depression symptom severity in children and adolescents. Each item consists of three sentences describing different levels of depressive symptom severity, and participants are asked to pick the option that best describes their feelings in the past two weeks. Each item is scored from 0 to 2, where higher scores indicate higher intensity of depression severity. The CDI-SF has demonstrated good convergent and divergent validity in children [62] and comparable sensitivity to the original CDI [63].

#### HPA axis regulation

The trier social stress test for children (TSST-C) [64] will be used to assess salivary cortisol reactivity and recovery to an acute stressor, thereby providing a measure of individual differences in HPA axis regulation. The TSST-C is a standardized procedure developed specifically for children and adolescents that reliably induces subjective stress and increases cortisol release [64]. A variant of the TSST-C has been validated for online use and is effective in eliciting a stress response in adolescents [65]. To control for diurnal fluctuations in cortisol levels, the videoconference session will be scheduled such that the first cortisol sample is collected between 3 and 6 PM. The task will begin with a baseline period during which participants will watch a calming nature video for 15 min. Participants will then be provided with an unfinished story as a prompt and given 5 min to prepare a speech. They will be told that their goal is to perform better than other adolescents their age. They will then present the story in a free speech in front of a panel of two adult judges (one female and one male) wearing lab coats and holding clipboards, while the videoconferencing session is recorded. The stressor task will take 10 min, during which participants will tell their story for 5 min and then do a mental arithmetic task for 5 min. Participants will then undergo a recovery period, during which they will first watch a 40-min calming nature video, and then complete a sociodemographic and medical history questionnaire derived from the Canadian Multicentre Osteoporosis Study (CaM*os*) [66]. To minimize repeated testing effects, a modified prompt [67] will be used in place of the story completion portion of the TSST-C in Wave 2. In Wave 3, the job interview prompt from the original TSST [34] will be used in place of the story completion. Different numbers will be used for the mental arithmetic portion in each Wave.

During the TSST-C, mood and cortisol reactivity will be assessed immediately after the baseline period, immediately before and after the stressor task, and four times during the recovery period: 10 min, 20 min, 30 min and 45 min after the stressor. Mood will be assessed using eight items adapted from The Positive and Negative Affect Schedule for Children (PANAS-C) [68] to assess the positive and negative emotions that participants experience throughout different time points during a social stressor task. Participants are asked to rate on a 5-point Likert scale (1 = *Very slightly or not at all* to *5* = *extremely*) the extent to which they currently feel happy, excited, calm, proud, stressed, upset, nervous and ashamed [69]. Biologically available (free) cortisol will be measured from saliva using the *Sarstedt* Salivette Cortisol collection tube (Sarstedt, Rommelsdorf, Germany). Participants will be asked to complete the Saliva Sampling Compliance Questionnaire prior to beginning the TSST-C procedure (see Additional file 2). For each saliva sample, a trained research assistant will instruct the participant, in accordance with Sarstedt’s established protocol for Salivette collection tubes (Sarstedt, Rommelsdorf, Germany), to roll a cotton swab around their mouth for 2 min before spitting it back into the tube. At the end of the TSST-C session, participants will be instructed to store their saliva samples in their home freezers until they mail it back to us, at which point the samples will be kept in a low-temperature freezer (− 30 °C or lower) until they are assayed for cortisol content.

At-home saliva sampling to assess diurnal cortisol variations will be done by collecting saliva samples using Salivettes and the instructions described above. As mentioned before, saliva sample collection will occur five times per day (immediately after wakening, 30 min after wakening, 45 min after wakening, 3 PM in the afternoon, and before bedtime) on two days in the early follicular phase of participants’ menstrual cycle. All samples will be assayed using a commercially available chemiluminescence immunoassay.

#### Social-emotional functioning

An emotion recognition task [70], adapted from Lischke et al. [71], will be used to assess socioemotional information processing. In this task, images of male and female faces displaying different emotional expressions, acquired from the NimStim Set of Facial Expressions (72), are presented in the center of a computer screen. In each trial, the face gradually morphs from a neutral to a full emotion expression (either sadness, fear, anger, or happiness). The appearance of a dynamically changing face is achieved by displaying, in sequence, 100 computer-generated still images transitioning from 0 to 100% emotion intensity. To avoid a perfect correlation between the passage of time and the intensity of the facial expression and to increase the difficulty of the task over time [70], some images are repeated (“jittered”) in the sequence. Any jitter could include between 2 and 5 repeats of the same image, and the total number of repeats allowed in each quartile of the progression was set to 10 to ensure a relatively even distribution of jitters. The resultant effect is of a face morphing slowly over the course of 35 s (100 unique primary images plus 40 jitters, each displayed for 250 ms [ms]). Participants are instructed to press the spacebar on the keyboard as soon as they recognize the displayed emotion. Upon the button press, participants receive an on-screen prompt to indicate which of the four emotions they detected. After completing two practice trials, participants undergo 2 blocks of 16 test trials, in which both blocks will use the same set of faces but will be presented in a different order and with different jitter patterns.

The child and adolescent social support scale (CASSS) [73] is a 60-item questionnaire that assesses children and adolescents’ perceived frequency and importance of social support from five key sources: parents, teachers, classmates, close friends and peers at school. For each source of support, 12 emotional, informational, instrumental and appraisal supportive behaviours are assessed for perceived frequency (1 = *never* to 6 = *always*) and importance (1 = *Not Important* to 3 = *Very Important*), where higher scores indicate higher frequency and importance of perceived support. Previous research has demonstrated strong psychometric properties for each source of support, including good internal consistency, test–retest reliability and construct validity in school-age children [73].

The UCLA loneliness scale (Version 3) [74] is a 20-item measure of feelings of loneliness. Participants are asked to rate how often each statement describes their own feelings on a 4-point Likert scale (1 = *Often* to 4 = *Never*), where higher scores indicate greater degrees of loneliness. The current and previous versions of this scale have been shown to have good internal consistency, test–retest reliability and validity in adults and adolescents [74, 75].

The ultimatum game [76], which will be presented on a computer interface, is played twice by each participant, once as a donor and once as a recipient. As the donor, the participant will begin the task with 100 points. They will be asked to choose how many points they are willing to offer to an anonymous recipient, with the knowledge that both they and the recipient will both receive 0 points if the recipient rejects the offer. If the recipient accepts the offer, both the recipient and donor will receive the agreed amount. Each participant will also be asked to indicate the lowest offer they are willing to accept from an anonymous donor, as a recipient. At the end of each wave, each participant will be randomly and anonymously matched with two other participants (one donor and one recipient) in the same wave. All players will then be allocated their points according to the responses they gave during the game. Participants’ offers as donors and acceptance threshold as recipients can elucidate their social decision-making behaviour and preferences for fairness [77].

#### Reward processing

The probabilistic reward task [45, 78] will be used to assess participants’ reward sensitivity. This task consists of 300 trials, divided into 3 blocks of 100 trials. Participants are told that the goal of the task is to win as much money as possible. At the beginning of each trial, participants will see a fixation cross in the center of the screen for 1400 ms. Depending on the wave of the study, participants will then see a mouthless or noseless cartoon face presented in the center of the screen for 500 ms, (Wave 1 and 3: mouthless, Wave 2: noseless). Next, in Waves 1 and 3, either a short mouth (10.00 mm) or a long mouth (11.00 mm) will be presented for 100 ms. In Wave 2, either a short nose (5.00 mm) or a long nose (5.31 mm) will be presented instead. Participants will be instructed to identify which stimulus (long or short) they detected by pressing the V or the M key on their keyboard (counterbalanced across participants). For each block, participants will be presented with the reward feedback of “Correct!! You won 20 cents” after 40 correct trials according to a controlled reinforcement schedule. Critically, one stimulus is programmed as the “rich stimuli”, and will be rewarded 3 times more frequently compared to the other stimulus, the “lean stimuli”, for correct responses. The association of “rich stimulus” with long vs short mouth (or nose) is counterbalanced across participants. At the end of the task, participants will earn $5.80 or $6.20 (counterbalanced across participants and waves).

#### Genetic variability

Each participant will be asked to provide a DNA sample using an Oragene 600 self-collection kit (DNA Genotek, Ottawa, Ontario, Canada) according to the manufacturer’s instructions. These kits allow for collection, long-term storage, and transportation of DNA at room temperature. DNA will be genotyped for over 750,000 variants on a custom genome-wide genotyping array (Illumina Global Screening Array) that captures both common and rare variation collected from large-scale sequencing projects and includes variants associated with multiple diseases and psychological/psychiatry-related variants.

#### Covariates and control variables

We will assess all confounds that, to our knowledge, have previously been found or proposed to at least partially account for the observed relationship between CHC use and depression, so that they can be accounted for in our analyses.

Sociodemographic information and medical history including but not limited to gender identity, sexual debut, history of menstruation, history of pregnancy and Body Mass Index (BMI; calculated using the formula: weight (kg)/height(m2)) will be collected. For gender identity and sexual debut questions, see Additional file 3.

The childhood trauma questionnaire-short form (CTQ-SF) [79] is a 28-item measure that screens for history of maltreatment and trauma in children and adolescents, including experiences of physical, sexual and emotional abuse as well as physical and emotional neglect. Questions are rated from 1 (*never true*) to 5 (*very often true*)*,* where higher scores indicate more experiences of childhood trauma*.* The scale has demonstrated good psychometric properties in adolescents [79]. This scale will be used to assess exposure to traumatic events in Wave 1.

The perceived stress scale (PSS) [80] is a 10-item scale that measures adolescents’ exposure to stress in the previous month. Items are scored on a 5-point Likert scale (1 = *Never* to 5 = *Very often),* where higher scores indicate greater levels of stress*.* Research in adolescents demonstrates the PSS’s good internal consistency, discriminant validity relative to intelligence, and concurrent validity relative to depression [81].

The medical outcomes study 36-item short-form health Survey (SF-36) [82] is a questionnaire that assesses health and quality of life. The measure contains subscales for physical functioning, role limitations due to physical health problems, role limitations due to personal or emotional problems, energy or fatigue, emotional well-being, social-emotional functioning, bodily pain and general health perceptions. It contains an additional item that measures perceived change in health in the past year. Each item is scored from 0 to 100, where a higher score indicates a better health state. This survey has good psychometric properties in adults [83], has been used successfully in research on adolescents [84, 85], and normative data on randomly sampled adolescents 16–19 are available from the local BC CaM*os* database [86].

Tanner staging [87] is a measure that assesses pubertal status in children and adolescents. In this study, we will use images of breast and pubic hair at different stages of sexual maturation [87, 88]. Participants are shown images depicting each Tanner stage of sexual maturation and asked to indicate which best represents their own level of maturation. A higher Tanner stage indicates more advanced pubertal development. The Tanner stages (0 to 5) have been shown to be positively associated with gonadal hormone levels in adolescents of both sexes [89] and demonstrates good validity in evaluating sexual maturation in children [90].

### Statistical analysis

First, in order to test whether CHC use is associated with increased likelihood of first diagnosis of MDD overall, we will use a logistic regression model on data collapsed across time points. We will adjust for calendar year (in part due to potential cohort effects as a result of the COVID-19 pandemic), as well as age at baseline, ethnicity, and socioeconomic status [12]. Using logistic regression, we will then calculate odds ratios and 95% CIs. We will also conduct a sensitivity analysis for young females who start using CHCs during the study, and will compare the odds of MDD for 6 months after the start of CHC use to the period before the start of CHC use.

Second, to specifically examine concurrent and prospective associations between CHC use and MDD at each time point, we will use a random intercept cross-lagged panel model (RI-CLPM). RI-CLPM assesses the chronological association between two outcome variables over several follow-ups, and models both within- and between-person effects [91]. By using RI-CLPM, we hope to assess the presence and direction of the association between CHC use and MDD diagnosis, over time. We will repeat this analysis to also assess the concurrent and prospective association between CHC use and, severity of MDD (mild, moderate, severe), as well as the association of CHC use and level of depressive symptoms, as assessed using the CDI-SF.

Third, we will use RI-CLPM to assess the prospective relationship between CHC use and several indicators of stress. We will first assess the prospective relationship between CHC use and responses to acute stress in the TSST-C. To assess responses to acute stress, we will calculate cortisol reactivity, as well as area under the curve with respect to the increase (AUC_I_) and area under the curve with respect to the ground (AUC_g_) [92]. Each participant’s cortisol reactivity will be calculated by subtracting the baseline cortisol level from the highest cortisol level obtained for that participant in that TSST-C [93]. AUC_I_ and AUC_g_ will be calculated using the trapezoid formulas described in [92]. We predict that CHC use will be associated with blunted cortisol reactivity and a smaller AUC_I_ in response to the TSST. Next, we will assess the prospective relationship between CHC use and diurnal cortisol slope (DCS), as well as the prospective relationship between CHC use and CAR. DCS will be defined as the slope of change in cortisol level from wakening to bedtime, and will be calculated as the slope of the line of best fit for the following samples: right after awakening, after school, bedtime [94]. CAR will be defined as the AUC_I_ and AUC_g_ of the following saliva samples: right after awakening, 30 min after awakening, and 45 min after awakening [95]. Given that mixed findings have been reported regarding the directions of association between CHC use, diurnal cortisol output (DCS and CAR), and depression, we consider these analyses exploratory in nature and do not specify directional hypotheses for these relationships.

Fourth, we will use the RI-CLPM to assess the prospective relation between CHC use and recognition of facial expressions of emotion. The dynamic morphing task used in this study is primarily designed to assess the intensity level at which a given participant is able to detect a specific emotion, and overall accuracy levels for this task are relatively high [70, 71]. Thus, we will assess the presence and direction of the association between CHC use and the intensity level on accurate trials at which participants are able to recognize facial expressions of emotion. We hypothesize that CHC users will demonstrate heightened sensitivity to sad faces (requiring lower intensities to identify them) and decreased sensitivity to happy faces (requiring higher intensities for identification).

Finally, to assess whether the predicted association of CHC use and depression (*i.e.*, depression level and MDD diagnosis) is mediated by changes in stress responses and/or emotion recognition, we will expand on the first two RI-CLPMs that assess the association of CHC use and MDD diagnosis, and the association of CHC use and depression level, by adding variables that assess stress response (*i.e.*, stress reactivity, AUC_I_, AUC_g_) and emotion recognition (*i.e.*, intensity of happiness and sadness required for accurate emotion recognition), separately, as mediators into these two models [96].

Additional exploratory analyses will address whether CHC use predicts changes in reward processing, social decision-making, self-reported loneliness, and perceived social support, and whether reward processing and/or social decision-making mediate the predicted relationship between CHC use and depression risk. We will also examine whether and how genetic variants involved in the uptake and metabolism of synthetic estrogen and progestin derivatives and/or previously linked to risk for depression moderate the predicted relationship between CHC use and depression risk.

To assess the robustness of our results, we will run a series of exploratory analyses to control for Tanner stages, SF-36 (Physical and Mental Component Summary Scores), sexual activity, SES, BMI, age of sexual debut, age at menarche, gender identity, PSS, and CTQ-SF, in all the RI-CLPM models. Given the complexity of the models and the number of possible covariates, in these exploratory analyses, we will use data-driven approaches to select covariates [97], and will make note of any findings to consider replicating in future confirmatory research.

### Power analysis

We used G*Power 3.1.9.7 [98, 99] to calculate the sample size necessary to address the primary research question: whether CHC use is associated with increased likelihood of first diagnosis of MDD. A total sample size of 290 participants was recommended by G*Power for using a binomial logistical regression model with *α* = 0.05, *Power* = 0.8, and odds ratio (OR) = 3.17. OR was calculated based on the Anderl et al. [15] findings showing that 16.1% of adolescent CHC users versus 5.7% of never users had experienced MDD in the previous year. To account for an expected 10% attrition rate, we aim to recruit 320 participants.

## Discussion

In this prospective cohort study, we will examine whether and how the use of CHC affects female adolescents’ risk for developing depressive symptoms, as well as for receiving a diagnosis of MDD. We will further explore potential links between CHC use and alterations in stress response and social-emotional functioning—two potential pathways through which CHC use may result in higher vulnerability for depression. As one of the first studies to assess the association between CHC use and depression in adolescents prospectively, our study has the potential to extend scientific knowledge about the relationship between adolescent CHC use and depression in several substantial ways. First, it will allow us to look at the temporal direction of the previously observed association between CHC use in adolescents and depression, that is, whether CHC precedes higher risk for depression, whether increased depression symptoms precede a higher likelihood of starting CHC use, or both.

Second, it will allow us to control for variables that have previously been found or proposed to at least partially account for the observed relationship between CHC use and depression, such as socioeconomic status, age of sexual debut, age of first CHC use, reasons for CHC use, and duration of CHC use [12, 14, 15, 100]. In previous studies assessing the relationship between CHC use and depression, the number and nature of control variables varied considerably, which may explain some of the apparent inconsistencies between findings.

Third, this study will specifically contribute to our understanding of the mechanisms through which CHC use in adolescents may increase the risk of depression. In particular, we aim to assess the effect of CHC use on the HPA axis and social-emotional functioning, two pathways through which CHC use might result in increased risk of depression. In addition to increasing scientific knowledge on the effect of CHC use on adolescents’ social-emotional development and risk of depression, this study is expected to provide the general public, especially adolescents and their parents, with essential information for making informed decisions about using CHC. In addition, understanding the nature and magnitude of CHC effects on adolescent development may help clinicians as they weigh the benefits and risks of prescribing CHCs to adolescents for both contraceptive and non-contraceptive (e.g., to control acne or regulate menstrual periods) reasons. Finally, by increasing our understanding of the risk of CHC use in adolescents, this study is expected to equip policymakers with necessary information to refine guidelines and recommendations for CHC use in adolescents. Ultimately, this line of research may reduce the incidence of depression in women, with positive downstream consequences for their families and communities.

## Supplementary Information

**Additional file 1.** Hormonal contraceptive use questionnaire. This questionnaire assesses participants’ history of hormonal contraceptive use, and includes questions about the current, past, and future plans to use of hormonal contraceptives, the age at the start of use, types of hormonal contraceptives used/being used, overall length of time of hormonal contraceptive use, and the reasons for using and discontinuing use of hormonal contraceptives.

**Additional file 2.** Saliva sampling compliance questionnaire. This questionnaire assesses participants’ compliance with the study instructions that aim to ensure the quality of saliva samples. In particular, this questionnaire asks participants whether they have ate, drank, or brushed their teath in the past hour, or have drank alcohol or used any nicotinic products in the past 12 hours.

**Additional file 3.** Gender identity and sexual debut questionnaire. This questionnaire assesses participants’ gender identity, sexual orientation, and their current and past sexual experinces, including the age of sexual debut and the number of current and past sexual partners.

**Additional file 4.** Consent form. This is a copy of the consent form that is given to the parents at Wave 1. This consent form includes information about the study procedures, risk and benefits associated with participating in the study, confidentiality, and the limits to confidentiality.

## Data Availability

Upon the completion of the study, data files will be stripped of any information that could identify the participants, and will be uploaded to an online form, or will be made available to other researchers upon reasonable request.

## Abbreviations

AUC_I_: Area under the curve with respect to the increase
AUC_g_: Area under the curve with respect to the ground
CAR: Cortisol awakening response
CASSS: The child and adolescent social support scale
CDI-SF: The children’s depression inventory – short form
CHC: Combined hormonal contraceptive
CTQ-SF: The childhood trauma questionnaire-short form
DCS: Diurnal cortisol slope
GAD: Generalized anxiety disorder
HPA: Hypothalamic pituitary adrenal
KSADS-PL: The kiddie schedule for affective disorders and schizophrenia for school-age children – present and lifetime
MDD: Major depressive disorder
OR: Odds ratio
PSS: The perceived stress scale
RI-CLPM: Random intercept cross-lagged panel model
SF-36: The Medical Outcomes Study 36-item short-form Health Survey
TSST: Trier social stress test
TSST-C: Trier social stress test for children
