# Supplementary material for: Assessing the role of adolescent hormonal contraceptive use on risk for depression: a 3-year longitudinal study protocol
Source: BMC Womens Health. 2022 Feb 23;22:48. doi: 10.1186/s12905-022-01623-2 (PMC8864455; doi:10.1186/s12905-022-01623-2)
Supplement: Supplementary file 1 — Additional file 1. Hormonal contraceptive use questionnaire. This questionnaire assesses participants’ history of hormonal contraceptive use, and includes questions about the current, past, and future plans to use of hormonal contraceptives, the age at the start of use, types of hormonal contraceptives used/being used, overall length of time of hormonal contraceptive use, and the reasons for using and discontinuing use of hormonal contraceptives. [file 12905_2022_1623_MOESM1_ESM.docx]

Hormonal Contraceptive Use Questionnaire

1. Have you ever used birth control pills or another type of hormonal contraceptives? (Note: This could be for *any*reason, such as birth control, period symptoms, acne, etc.)

- Yes
- No

2. [If no] Are you planning to start using birth control pills or another type of hormonal contraceptive in the next month? (Note: This could be for *any* reason, such as birth control, period symptoms, acne, etc.)

- Yes
- Maybe
- No

3. At what age did you start? (*approximately, in years) (If you were 12 years old, write: "12".)* ______________________________________

4. What type(s) of hormonal contraceptives have you used (if you have used more than one type, please list all of them)?
______________________________________

5. Not counting any time when you stopped taking them, for how long **altogether** have you taken birth control pills or another type of hormonal contraceptives? *(approx, in years & months) (If you have taken birth control pills for 0 years and 3 months, write "0y 3m".)*______________________________________

6. Are you still using birth control pills or another type of hormonal contraceptive?

- Yes
- No

7. [If no] At what age did you stop using birth control pills or another type of hormonal contraceptive? *(in years)* (If you were 12 years old, write: "12".)

______________________________________

8. [If no] Why did you stop using birth control pills or another type of hormonal contraceptive?

______________________________________

9. Which of the following was the main reason for which you first used birth control pills or another type of hormonal contraceptives?

- To prevent pregnancy. (1)
- To treat premenstrual symptoms. (2)
- To treat heavy menstrual flow or abnormal bleeding. (3)
- To treat severe menstrual cramps (dysmenorrhea). (4)
- To treat irregular or infrequent periods. (5)
- To treat acne or unwanted facial or body hair. (6)

10. Were there any other reasons for which you first used birth control pills or another type of hormonal contraceptives?

- To prevent pregnancy. (1)
- To treat premenstrual symptoms. (2)
- To treat heavy menstrual flow or abnormal bleeding. (3)
- To treat severe menstrual cramps (dysmenorrhea). (4)
- To treat irregular or infrequent periods. (5)
- To treat acne or unwanted facial or body hair. (6)
