# Supplementary material for: Assessing the role of adolescent hormonal contraceptive use on risk for depression: a 3-year longitudinal study protocol
Source: BMC Womens Health. 2022 Feb 23;22:48. doi: 10.1186/s12905-022-01623-2 (PMC8864455; doi:10.1186/s12905-022-01623-2)
Supplement: Supplementary file 2 — Additional file 2. Saliva sampling compliance questionnaire. This questionnaire assesses participants’ compliance with the study instructions that aim to ensure the quality of saliva samples. In particular, this questionnaire asks participants whether they have ate, drank, or brushed their teath in the past hour, or have drank alcohol or used any nicotinic products in the past 12 hours. [file 12905_2022_1623_MOESM2_ESM.docx]

Saliva Sampling Compliance Questionnaire

Please answer each of these questions completely and honestly. We understand that mistakes happen, and participants are not always able to collect saliva perfectly. 

However, it is important that you let us know if anything unusual happens. This information will be very important for us when we analyze your saliva sample. Thank you!

11. Did you drink anything in the past hour?

- Yes
- No

12. What did you drink, and how much?

________________________________________________________________

13. How long ago was it that you drank something? [in minutes]

________________________________________________________________

14. Did you eat anything in the past hour?

- Yes
- No

15. What did you eat, and how much?

________________________________________________________________

16. How long ago was it that you ate something? [in minutes]

________________________________________________________________

17. Did you brush your teeth in the past hour?

- Yes
- No

18. Did you drink any alcoholic beverages in the past 12 hours?

- Yes
- No

19. What alcoholic beverage(s) did you drink, and how much?

________________________________________________________________

20. How long ago was it that you drank an alcoholic beverage? [in hours]

________________________________________________________________

21. Did you drink any caffeinated beverages (e.g., black tea, green tea, coffee, energy drinks) in the past 12 hours?

- Yes
- No

22. What caffeinated beverage(s) did you drink, and how much?

________________________________________________________________

23. How long ago was it that you drank a caffeinated beverage? [in hours]

________________________________________________________________

24. Have you used any products containing nicotine in the past 12 hours?

- Yes -- cigarettes
- Yes -- electronic nicotine product (ex. e-cigarettes, vapes, etc.)
- Yes -- other (specify) ____________________________________________
- No

25. How many cigarettes did you smoke?

________________________________________________________________

26. How long ago was it that you last smoked a cigarette? [in hours]

________________________________________________________________

27. What concentration of nicotine e-juice or e-liquid did you use?

- (in mg/mL) ____________________________________________
- (in %) ________________________________________________
- I don't know.

28. Approximately how much nicotine e-juice or e-liquid did you use? (in mL)

________________________________________________________________

29. How long ago was it that you last used an electronic nicotine product (ex. e-cigarette, vape, etc.)? [in hours]

________________________________________________________________

30. Approximately how much nicotine did you use from some "other" nicotine product?

________________________________________________________________

31. How long ago was it that you last used some "other" nicotine product? [in hours]

________________________________________________________________

32. Please answer this question completely and honestly. We understand that mistakes happen, and participants are not always able to collect saliva perfectly. However, it is important that you let us know if anything unusual happens. This information will be very important for us when we analyze your saliva sample. Thank you!

Did anything unusual happen?

Here are some examples of what you could mention:

a. I collected the sample a little late. It was supposed to be collected at 8:30 am when I woke up, but I collected it at 9:30 am instead.
b. At first, I accidentally used the wrong tube (labelled 000PA2) so I rinsed it out with water and put it back in its bag. Then I used the right tube.

________________________________________________________________
