# Supplementary material for: Assessing the role of adolescent hormonal contraceptive use on risk for depression: a 3-year longitudinal study protocol
Source: BMC Womens Health. 2022 Feb 23;22:48. doi: 10.1186/s12905-022-01623-2 (PMC8864455; doi:10.1186/s12905-022-01623-2)
Supplement: Supplementary file 3 — Additional file 3. Gender identity and sexual debut questionnaire. This questionnaire assesses participants’ gender identity, sexual orientation, and their current and past sexual experinces, including the age of sexual debut and the number of current and past sexual partners. [file 12905_2022_1623_MOESM3_ESM.docx]

Gender Identity Questionnaire

251. Sex is what a person is born. Gender is how a person feels. When a person's sex and gender do not match, they might think of themselves as transgender. Which one response best describes you?

- I am not transgender. (1)
- I am transgender and identify as a boy or man. (2)
- I am transgender and identify in some other way (such as non-binary, genderqueer, or gender fluid). (3)
- I am not sure if I am transgender. (4)
- I do not know what this question is asking. (5)

Sexual Debut

242. The next set of questions is about your sexual behavior. By sex, we mean vaginal, oral, or anal sex. Please remember that your answers are strictly confidential.

______________________________________

243. Have you ever had sex?

- Yes (1)
- No (3)

244. [If yes] How old were you when you had sex for the first time?
(For example, if you were 13 years old, write: "13".)

________________________________________________________________

245. [If yes] In your lifetime, with how many boys/men have you had sex?

________________________________________________________________

246. [If yes] In the past 12 months, with how many boys/men have you had sex?

________________________________________________________________

247. [If yes] In your lifetime, with how many girls/women have you had sex?

________________________________________________________________

248. [If yes] In the past 12 months, with how many girls/women have you had sex?

________________________________________________________________

If Sex is what a person is born. Gender is how a person feels. When a person's sex and gender do not... = I am not transgender.

And Sex is what a person is born. Gender is how a person feels. When a person's sex and gender do not... = I do not know what this question is asking.

249. Which of the following best describes your feelings?

- Completely heterosexual/straight (only attracted to boys/men) (1)
- Mostly heterosexual/straight (primarily attracted to boys/men, but also somewhat attracted to girls/women) (2)
- Bisexual (equally attracted to girls/women and boys/men) (3)
- Mostly homosexual/lesbian (primarily attracted to girls/women, but also somewhat attracted to boys/men) (4)
- Completely homosexual/lesbian (only attracted to girls/women) (5)
- Not sure/questioning (6)
- Other (please specify): (7) ________________________________________________

If Sex is what a person is born. Gender is how a person feels. When a person's sex and gender do not... = I am not transgender.

Or Sex is what a person is born. Gender is how a person feels. When a person's sex and gender do not... = I do not know what this question is asking.

252. Which of the following best describes your feelings?

- Only attracted to boys/men (1)
- Primarily attracted to boys/men, but also somewhat attracted to girls/women (2)
- Equally attracted to girls/women and boys/men (3)
- Primarily attracted to girls/women, but also somewhat attracted to boys/men (4)
- Only attracted to girls/women (5)
- Not sure/questioning (6)
- Other (please specify): (7) ________________________________________________
