# Supplementary material for: Assessing the role of adolescent hormonal contraceptive use on risk for depression: a 3-year longitudinal study protocol
Source: BMC Womens Health. 2022 Feb 23;22:48. doi: 10.1186/s12905-022-01623-2 (PMC8864455; doi:10.1186/s12905-022-01623-2)
Supplement: Supplementary file 4 — Additional file 4. Consent form. This is a copy of the consent form that is given to the parents at Wave 1. This consent form includes information about the study procedures, risk and benefits associated with participating in the study, confidentiality, and the limits to confidentiality. [file 12905_2022_1623_MOESM4_ESM.docx]

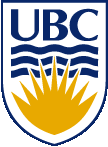


**Department of Psychology**

**University of British Columbia**

**Vancouver, BC, V6T 1Z4**

Phone: 604.822.2755

Fax: 604.822.6923

The University of British Columbia

**Consent Form**

**Research Project Title: UBC Teen Health and Emotions Study**

**Principal Investigator**: Dr. Frances Chen, Assistant Professor, UBC Department of Psychology

**Co-Principal Investigator**: Dr. Joelle LeMoult, Assistant Professor, UBC Department of Psychology

**Co-Investigators**:

Dr. Christine Anderl, Postdoctoral Fellow, UBC Department of Psychology

Dr. Liisa Galea, Professor, UBC Department of Psychology

Dr. Jerilynn C. Prior, Professor, UBC Department of Endocrinology and Metabolism

Dr. Colin Ross, Assistant Professor, UBC Department of Pharmacogenomics

Bita Zareian, Graduate Student, UBC Department of Psychology

**Introduction and Purpose:** In this study we are interested in how sex hormones (both hormones that are naturally produced by the body and hormones that are included in specific types of medication such as birth control pills) affect young women’s emotions, social behaviour, and mental health. This research is funded by a project grant from the Canadian Institutes of Health Research.

**Study Procedures:** This consent form describes what will happen to you and your child during the study; it will also describe any possible benefits, risks, and discomforts. Before making a decision whether to participate or not, please read the following information carefully. If you and your child agree to participate in this study, this is what would happen:

| First Session | Second Session |
| --- | --- |
| - Interview - Fill out some questionnaires - Computerized task - Saliva sample | - Fill out some questionnaires - Computerized tasks - Telling a story & solving math problems - Saliva samples |

Firstly, prior to your first session at Wave 1, you will receive a package containing saliva sampling tubes, a DNA testing kit, and a tape measure. At Wave 1, your child will be invited to an online Zoom session to complete an interview about her past experience with stress as well as on their feelings and behaviours. One example question is, “do you ever feel down, blue or depressed?” We will also ask your child to fill out some questionnaires about their experiences, feelings, thoughts, and behaviours. For example, she would be asked about the support she receives from friends and how she copes with problems. These activities will approximately take 2.5 hours in total. We will also ask you about your child’s feelings, behaviours, and experiences, and we will ask about your family’s background (for example, your family income and your occupation). This interview will take approximately 30 to 60 minutes. You and your child will complete these activities in parallel, but separately. Finally, your child will also be asked to give a saliva sample for genetic testing.

Your child will also be asked to complete at-home hormone saliva sampling on two consecutive days to measure a stress hormone called cortisol. On the days of collection, your child will collect saliva five times in the day: three times in the morning, at mid-afternoon, and in the evening.

Lastly, at Wave 1, your child will be invited to a second Zoom session to complete the following computer and interactive tasks as well as a brief questionnaire:

- Your child will also be asked to view a set of photographed faces and to try to recognize the emotions they display.
- Your child will be asked to tell a story in front of a small audience and solve some math problems. Your child will also be asked to provide saliva samples before, during and after the story-telling task, which will later be tested for hormones.
- Your child will be asked questions about her medical and dietary history.

Genetic testing will be performed in the laboratory of study co-investigator Dr. Colin Ross, Assistant Professor at the UBC Department of Pharmacogenomics. In particular, he will perform a test (a “GWAS”, or “genome-wide association study”) to identify which versions of different genes your child is carrying. We are particularly interested in genetic variants regulating the liver enzyme CYP (CYP3A/4), estrogen receptors (ESR1/2), and mineralcorticoid receptors (NR3C2), because these can influence how hormones are processed in the body. Beyond the testing identified, no other genetic analysis will be performed. Potential findings that suggest participants might have undiagnosed conditions will not be validated by the research team and the individual results of these analyses will not be returned to our study participants.

We will repeat the above procedures 18 months (Wave 2) and 36 months (Wave 3) after the initial session.

To determine your child's eligibility for future sessions, we are looking at a pattern of responses. The main criteria that we will take into consideration in determining your child's eligibility for the study are:

- Your child is female and currently age 13-15 years
- Your child has already had her first menstrual period and was 10 years or older at her first menstrual period
- Your child does not have symptoms consistent with a substance use disorder, mania, or psychosis, or severe impairment caused by a head trauma
- Your child does not / did not previously have a condition that is known to influence sex hormone levels (such as an endocrine disorder or endometriosis) and does / did not take any medication that alters sex hormone levels
- You and your child’s participation in this study does not result in any dual relationship with the researchers
- You and your child are fluent in English

**Confidentiality:** You and your child’s confidentiality will be respected and protected. We will ask you and your child to use a code name, rather than your own names, to sign on Zoom. You will also be allowed to turn off your camera and microphone when we do not need your video and audio during the session. All of the information that you and your child provide in the context of this study will be kept strictly confidential within the research team. This means that we will also not share any information that you provide with your child or any information that your child provides with you. However, if we believe that you are going to harm yourself or others, or if we believe that a child is being harmed or is at risk of being harmed, we may need to break confidentiality in order to keep you or others safe. This may include consulting with a Psychologist to provide feedback and/or resources. Biological, interview, video, and computer data that is collected will not be linked to your name or your child’s name, but rather to a code number that will be assigned to your child for the duration of the study. Furthermore, this information will be kept secure (e.g., in an encrypted file on a password-protected server located in UBC psychology building or on a secure server hosted by UBC (OneDrive)). Access to all data collected during the study will be restricted to Dr. Chen and her research team at the University of British Columbia (UBC). Once the study is completed, you may request a summary of these results from the principal investigator. This summary will not provide any identifying information about you/your child or any other research participants. Electronic data will be preserved for future use in open science initiatives, which allow for researchers from different universities to share their data upon completion of studies, in an effort to stimulate exploration of existing data sets. Data files are uploaded to an online form, and these files are stripped of any information that could identify you or your child (e.g., names) to ensure confidentiality and anonymity.

The web survey company servers record incoming IP addresses of the computer that you use to access the survey but no connection is made between your/your child’s data and the computer’s IP address. We are using an online server company that stores all customer data in a specific location; it does not float around in the “cloud.” In addition, all data are processed in Canada, and are not moved to another jurisdictional area. The security and privacy policy for the web survey company can be provided to you by contacting the research team.

**Potential Risks of Participation:** It is possible that participating in this study could trigger an emotional/stress response in your child, although we believe that the risk is very low in both probability and severity. You and your child can quit the study at any time and you are both free to decide not to answer any particular question. If you or your child decide to withdraw from the study, all of your data will be destroyed.

**Potential Benefits of Participation:** There are no direct benefits of participating. Indirectly, you may gain a better understanding of psychological research, as well as an appreciation that your participation will help to further the scientific understanding of the variables of interest.

**Remuneration:** At the end of each laboratory test session, participating families will receive an honorarium of $30, which will be prorated at a rate of $7.50/30 minutes if you only complete a portion of the study procedures.

In addition, during each wave, participants will have the chance to win between $5-11 based on their performance on two of the study tasks. In session 1, they will receive up to $6 depending on their responses to one of the computerized tasks. In session 2, they will have the chance to receive up to $5 which will depend on their own decisions as well as the decisions of their randomly selected partner (another participant) in one of the interactive computer tasks.

**Contact Information for the Study:** This study is being conducted by Dr. Frances Chen, the Principal Investigator, with funding from the Canadian Institutes of Health Research (CIHR). If you have any questions, please contact Dr. Frances Chen or our lab at 604-822-9957 or socialhealthlab@psych.ubc.ca.

**Contact for Concerns about the Rights of Research Subjects:** If you have any concerns or complaints about your rights as a research participant and/or your experiences while participating in this study, contact the Research Participant Complaint Line in the UBC Office of Research Ethics at 604-822-8598, or if long distance, [RSIL@ors.ubc.ca](mailto:RSIL@ors.ubc.ca) or 1-877-822-8598.

**Consent:** Participation is entirely voluntary, and you may withdraw your consent for any reason and at any time you wish to do so. You may also not answer any question for any reason.

Please check off all activities to which you are consenting to participate:

- You and your child’s participation in Wave 1 activities
- You and your child’s participation in Wave 2 activities
- You and your child’s participation in Wave 3 activities

Even though you are consenting to these activities now, you or your child may later choose to not participate in any specific activity or to withdraw your consent entirely.

If you would like you and your child to participate in this study, please choose the option “I consent for me and my child to participate in this study”, and type your full legal name in the space below. If you do not want you and your child to participate in this study, please choose the option “I do not wish for me and my child to participate in this study.”

- I consent for me and my child to participate in this study
- I do not wish for me and my child to participate in this study
